# Chimeric peptide EP45 as a dual agonist at GLP-1 and NPY2R receptors

**DOI:** 10.1038/s41598-018-22106-1

**Published:** 2018-02-28

**Authors:** Oleg G. Chepurny, Ron L. Bonaccorso, Colin A. Leech, Torsten Wöllert, George M. Langford, Frank Schwede, Christian L. Roth, Robert P. Doyle, George G. Holz

**Affiliations:** 1 Department of Medicine, State University of New York (SUNY) Upstate Medical University, 505 Irving Avenue, Syracuse, NY 13210 USA; 2 Department of Chemistry, Syracuse University, 111 College Place, Syracuse, NY 13244 USA; 3 Department of Biology, Syracuse University, Syracuse, NY 13244 USA; 4 BIOLOG Life Science Institute, 28199 Bremen, Germany; 5 Center for Integrative Brain Research, Seattle Children’s Research Institute, Washington, 98105 USA; 6 Department of Pediatrics, University of Washington, Seattle, Washington 98105 USA; 7 Department of Pharmacology, State University of New York (SUNY) Upstate Medical University, 505 Irving Avenue, Syracuse, NY 13210 USA

## Abstract

We report the design and target validation of chimeric peptide EP45, a novel 45 amino acid monomeric dual agonist peptide that contains amino acid sequence motifs present within the blood glucose-lowering agent exendin-4 (Ex-4) and the appetite-suppressing agent PYY(3–36). In a new high-throughput FRET assay that provides real-time kinetic information concerning levels of cAMP in living cells, EP45 recapitulates the action of Ex-4 to stimulate cAMP production via the glucagon-like peptide-1 receptor (GLP-1R), while also recapitulating the action of PYY(3–36) to inhibit cAMP production via the neuropeptide Y_2_ receptor (NPY2R). EP45 fails to activate glucagon or GIP receptors, whereas for cells that co-express NPY2R and adenosine A_2B_ receptors, EP45 acts in an NPY2R-mediated manner to suppress stimulatory effects of adenosine on cAMP production. Collectively, such findings are remarkable in that they suggest a new strategy in which the co-existing metabolic disorders of type 2 diabetes and obesity will be treatable using a single peptide such as EP45 that lowers levels of blood glucose by virtue of its GLP-1R-mediated effect, while simultaneously suppressing appetite by virtue of its NPY2R-mediated effect.

## Introduction

The concept of multi-purpose peptide drugs, efficacious for the simultaneous treatment of several disease states, has become the focus of intense biomedical research in recent years. Drawing on the sequences of more than 7,000 naturally occurring peptides with important biological functions in humans, new dual agonist peptides can be designed so that they have the capacity to stimulate two different G protein-coupled receptors (GPCRs)^1–3^. These dual agonist peptides have expanded biological functions owing to their dual receptor specificity^4^. Here, we report the design and target validation of EP45, a chimeric dual agonist peptide that has the surprising ability to act as an agonist at both the glucagon-like peptide-1 receptor (GLP-1R) and the neuropeptide Y_2_ receptor (NPY2R). These two GPCRs activated by EP45 are of major importance to metabolic homeostasis in humans^5–10^, and they are both established targets for drug discovery research that is relevant to the treatment of type 2 diabetes (T2DM) and obesity^5,6,11–13^.

Chimeric peptides contain amino acid motifs found within two different peptides^14^. This chimeric structure distinguishes them from a separate class of dual- or tri- agonist molecules in which conjugation strategies involving synthetic linker chemistry are used to fuse the full-length amino acid sequences of peptides such as the hormones glucagon, GLP-1, gastrin, and gastric inhibitory polypeptide (GIP)^1–3,15^. Increasingly, it is recognized that appropriately designed chimeric peptides are of clinical relevance in that they offer pharmacological advantages over full-length conjugated peptides^16^. These advantages include improved pharmacokinetics and bioavailability *in vivo*, as well as the ability to fine-tune dual agonist potency and efficacy through selective modifications of amino acid residues that are important to GPCR activation.

The worldwide prevalence of obesity, diabetes, and associated metabolic complications increase the risk of cardiovascular disease and stroke, which collectively present a great threat to public health^17,18^. New strategies designed to combat these disorders take advantage of the blood glucose-lowering and appetite-suppressing properties of GLP-1 and PYY(3–36), two peptides that are co-released from intestinal L-cells in response to ingested nutrients^19,20^. GLP-1 and its structurally-related synthetic peptides (e.g., exenatide, liraglutide) lower levels of blood glucose in patients with T2DM while also suppressing appetite in some of these patients^5,6,21–23^. PYY(3–36) is currently under investigation for use in the treatment of obesity, and it also suppresses appetite^4,9,13,24^ Interestingly, exaggerated secretion of GLP-1 and PYY(3–36) occurs in some gastric bypass (RYGB) surgery patients, and these alterations correlate with long-term appetite suppression and weight-loss that is associated with such surgery^25^.

GLP-1 binds to the GLP-1R, a Class B GPCR that signals through G_S_ proteins to stimulate cAMP production^26^, and that is expressed not only on pancreatic beta cells where it regulates insulin secretion^27,28^, but also on hypothalamic and vagal sensory neurons where it regulates appetite^29^. PYY(3–36) instead binds to NPY2R, a Class A GPCR that signals through G_i_ proteins to inhibit cAMP production, and that also participates in the hypothalamic control of appetite^10,29^. Here, we report novel dual agonist properties of EP45 at the GLP-1R and NPY2R, as revealed using a new high-throughput fluorescence resonance energy transfer (FRET) assay^30,31^. Such dual agonist properties of EP45 are understandable since its design incorporates amino acid motifs present within the GLP-1R agonist exendin-4, and also the NPY2R agonist PYY(3–36)^32,33^. Since the GLP-1R and NPY2R differ with respect to their tissue distribution and signaling properties, a dual agonist peptide such as EP45 might constitute an ideal medicinal agent with which to simultaneously treat T2DM and obesity.

## Results

### A molecular tool kit for studies of EP45 dual agonist action

**Figure 1:**
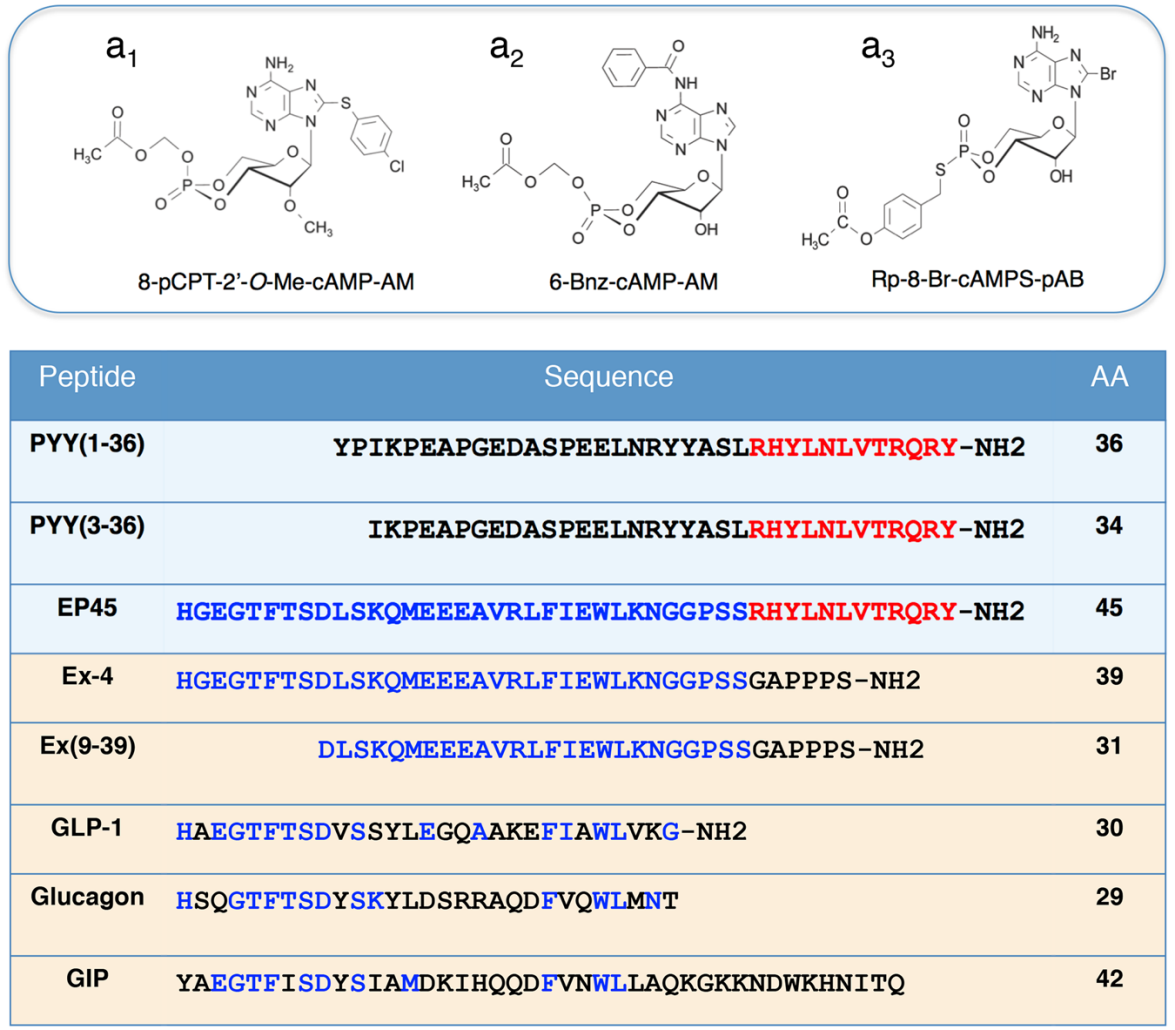
cAMP analog structures and peptide sequence alignments. 8-pCPT-2′-*O*-Me-cAMP-AM (**a**_**1**_) and 6-Bnz-cAMP (**a**_**2**_) are selective activators of Epac and PKA, respectively. Rp-8-Br-cAMPS-pAB (**a**_**3**_) is an antagonist of Epac and PKA activation. Color-coding of peptides shown below in red indicates amino acid residues within EP45 that correspond to residues present in PYY(1–36) or PYY(3–36). Color-coding in blue indicates amino acid residues within EP45 that correspond to residues present in the GLP-1R agonist exendin-4 (Ex-4) or the GLP-1R antagonist exendin(9–39) abbreviated as Ex(9–39), or in the hormones GLP-1, glucagon, and GIP. Abbreviation: AA, amino acid chain length.

We established a new high-throughput FRET assay for detection of cAMP using our HEK293-H188-C24 cell line that stably expresses the cAMP biosensor H188 developed by Klarenbeek and co-workers^30^. Since this FRET assay is broadly applicable to cyclic nucleotide research^31^, we first tested the sensitivity, dynamic range, and cAMP responsiveness of H188 using highly membrane permeable cAMP agonists that included the acetoxymethyl esters (AM-esters) of Epac (8-pCPT-2′-*O*-Me-cAMP-AM) or PKA (6-Bnz-cAMP-AM) selective cAMP analogs (Fig. 1)^34,35^. Also tested was Rp-8-Br-cAMPS-pAB, a highly membrane permeable para-acetoxybenzyl ester (pAB-ester) prodrug of the cAMP antagonist Rp-8-Br-cAMPS (Fig. 1)^36^. Using forskolin that stimulates cAMP production, or the cyclic nucleotide phosphodiesterase (PDE) inhibitor isobutylmethylxanthine (IBMX) that slows cAMP degradation, the capacity of H188 to detect cAMP was also determined. The immediate aim of these investigations was to validate a microplate reader assay that allowed live-cell detection of cAMP in a 96-well format^31^. This FRET assay was then used to evaluate how levels of cAMP were altered by PYY(1–36), PYY(3–36), exendin-4, exendin(9–39), GLP-1, glucagon, and GIP (Fig. 1). Using rational drug design, we synthesized and tested the 45 amino acid monomeric chimeric peptide EP45 that contains the partial amino acid sequences of exendin-4 and PYY(3–36) at its N- and C-termini, respectively (Fig. 1). As described in greater detail below for cells expressing recombinant GPCRs, EP45 acted as an agonist at the GLP-1R to stimulate cAMP production, whereas it instead inhibited cAMP production by acting as an agonist at NPY2R. Thus, novel dual agonist properties of EP45 were revealed by this FRET assay.

### High-throughput FRET assays for detection of EP45 dual agonist action

**Figure 2:**
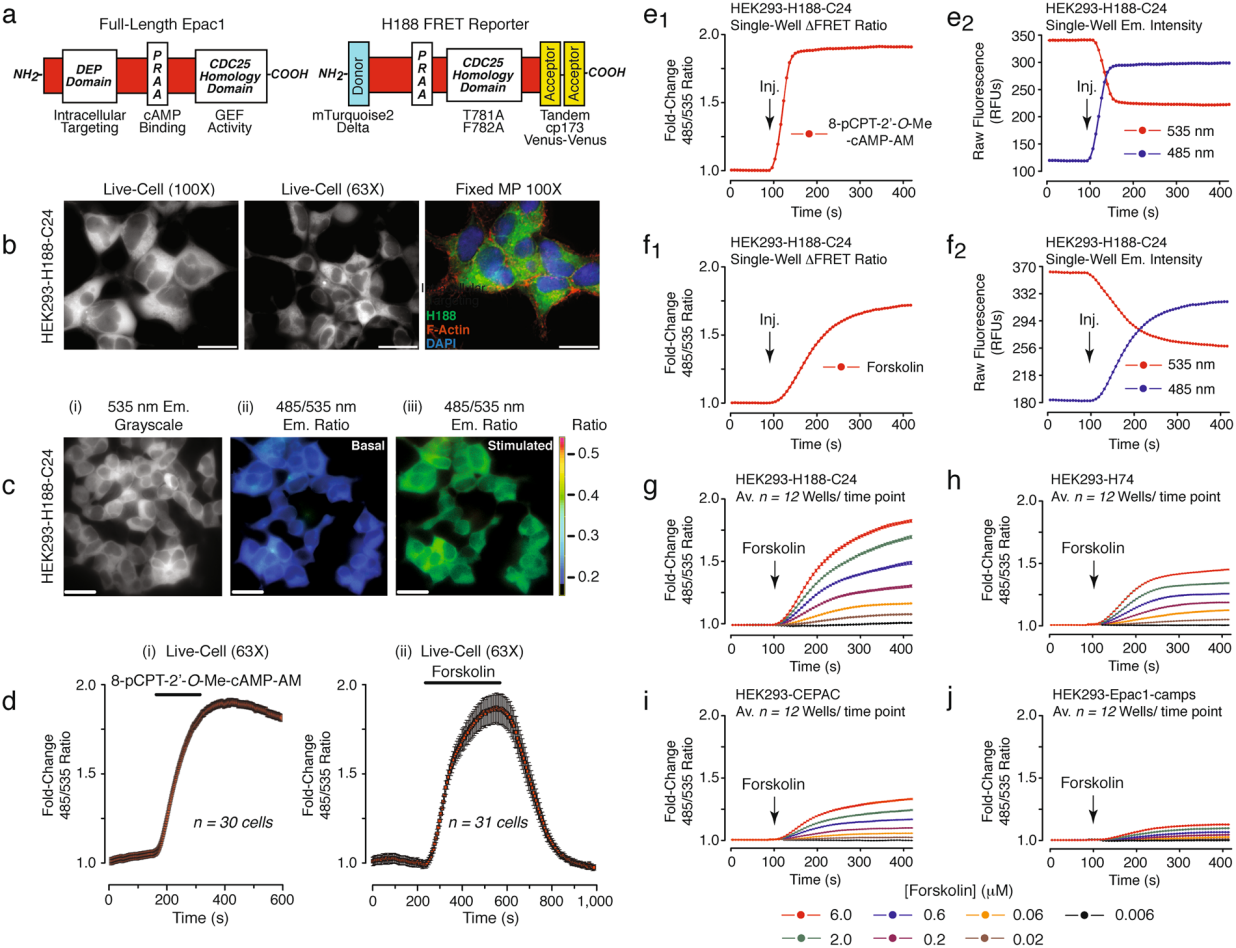
HEK293-H188-C24 cells for FRET-based detection of cAMP. (**a**) Domain structures of Epac1 (left) and H188 (right). (**b**) Live-cell imaging at 100x (left panel) and 63x (middle panel) magnification using a YFP filter set illustrates H188 fluorescence in HEK293-H188-C24 cells. Major projection (MP) overlays of Z-stack images (right panel) illustrate the distribution of cytosolic H188 fluorescence, cytoskeletal F-actin immunoreactivity, and DAPI nuclear staining. Calibration bars: 10 μm for 100x; 20 μm for 63x. (**c**) FRET-based live-cell imaging illustrates the 535 nm emission fluorescence (i) and the 485/535 nm FRET ratio (ii,iii) prior to (i,ii) and during (iii) exposure of cells to 10 μM 8-pCPT-2′-*O*-Me-cAMP-AM. Calibration bars: 20 μm. (**d**) Time course of the ΔFRET measured prior to, during (horizontal bars), and after exposure of cells to 10 μM 8-pCPT-2′-*O*-Me-cAMP-AM (i) or forskolin (ii) applied by bath superfusion. Error bars indicate the mean+/− s.e.m for *n* = *30 cells* (i) or *n* = *31 cells* (ii). *Y-axis* values are expressed as the fold-change of baseline-subtracted 485/535 nm FRET ratios so that a value of 1 corresponds to the baseline ratio, and a value of 2 corresponds to a doubling of the ratio, here and in all subsequent figures. (**e1**,**e2**) Single-well detection of ΔFRET (**e**_**1**_) and 485 or 535 nm H188 raw fluorescence unit (RFU) emission (Em.) intensity (**e**_**2**_) measured in response to injection (Inj., arrow) of 8-pCPT-2′-*O*-Me-cAMP-AM (50 μl; 10 μM final concentration) for HEK293-H188-C24 cell monolayers pre-equilibrated in 200 μl of saline. (**f**_**1**_,**f**_**2**_) Same as in **e**_**1**_/**e**_**2**_ except that the test solution contained forskolin at a 2 μM final concentration. (**g–j**) HEK293 cells stably expressing H188 (**g**), H74 (**h**), CEPAC (**i**), or Epac1-camps (**j**) were administered forskolin (0.006–6.0 μM final concentrations).

H188 contains the cAMP sensor Epac1 in which the full-length protein is truncated to remove the Disheveled, Egl-10, and Pleckstrin (DEP) membrane-targeting domain so that its expression is confined largely to the cytoplasm (Fig. 2a,b)^30^. T781A and F782A mutations introduced within the CDC25 homology domain disrupt guanine nucleotide exchange factor (GEF) activity so that binding of cAMP to H188 is not linked to downstream signaling events mediated by the Epac1 effector protein Rap1 GTPase (Fig. 2a)^30^. In H188 the Epac1 backbone is flanked by an mTurquoise2Δ FRET donor chromophore at the N-terminus, and tandem cp173 Venus-Venus FRET acceptor chromophores at the C-terminus (Fig. 2a). Binding of cAMP reduces FRET so that when exciting with 440 nm light, there is an increase of 485 nm (mTuquoise2Δ) and a decrease of 535 nm (cp173 Venus-Venus) emission fluorescence. This ΔFRET is measured as an increase of the 485/535 nm FRET ratio.

The action of cAMP to reduce H188 FRET was confirmed by fluorescence microscopy using HEK293-H188-C24 cells grown on glass coverslips. In these single-cell assays, the expected ΔFRET was measured after exposure of cells to the selective Epac activator 8-pCPT-2′-*O*-Me-cAMP-AM or the cAMP-elevating agent forskolin (Fig. 2c,d). The ΔFRET was also detectable in single wells of a 96-well plate when using a microplate reader and monolayers of HEK293-H188-C24 cells. Thus, for cells treated with 8-pCPT-2′-*O*-Me-cAMP-AM or forskolin, reciprocal changes of the 485 and 535 nm emission intensities were measured with high precision (Fig. 2e1,e2,f1,f2). H188 exhibited wide dynamic range (2-fold ΔFRET), high sensitivity (EC_50_ 500 nM), and excellent Z-score (0.77) when testing forskolin. Live-cell calibrations using digitonin-permeabilized cells indicated that a 2-fold ΔFRET occurred over a concentration range of 3–1,000 μM cAMP (Suppl. Figure 1).

**Figure 3:**
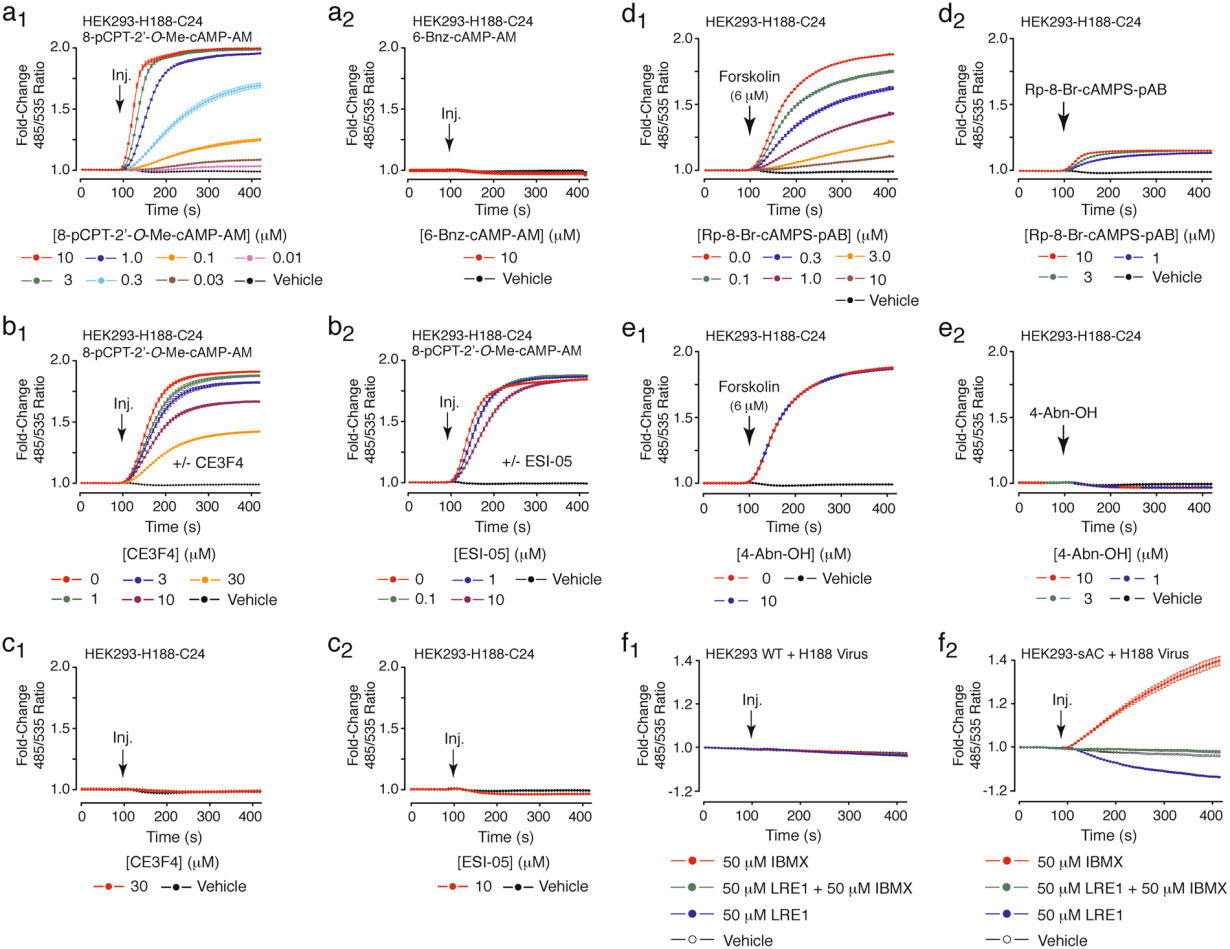
HEK293-H188-C24 cells provide a new platform for drug discovery. (**a**_**1**_,**a**_**2**_) H188 expressed in HEK293-H188-C24 cells responded to the Epac activator 8-pCPT-2′-*O*-Me-cAMP-AM (**a**_**1**_), but not the PKA activator 6-Bnz-cAMP-AM (**a**_**2**_). (**b**_**1**_,**b**_**2**_) The action of 8-pCPT-2′-*O*-Me-cAMP-AM was reduced by the Epac1 inhibitor CE3F4 (**b**_**1**_), but not the Epac2 inhibitor ESI-05 (**b**_**2**_). (**c**_**1**_,**c**_**2**_) The baseline FRET ratio was unaffected by CE3F4 (**c**_**1**_) or ESI-05 (**c**_**2**_). (**d**_**1**_,**d**_**2**_) Rp-8-Br-cAMPS-pAB blocked the action of forskolin at H188 (**d**_**1**_) while also exerting a weak stimulatory effect when it was administered alone (**d**_**2**_). (**e**_**1**_,**e**_**2**_) The action of forskolin was not blocked by the negative control 4-acetoxybenzy alcohol (4-Abn-OH) that lacks the cAMP moiety (**e**_**1**_), nor did 4-Abn-OH alter the baseline FRET ratio (**e**_**2**_). Note that cytosolic esterases hydrolyze Rp-8-Br-cAMPS-pAB and 4-Abn-OH to generate the metabolites 4-hydroxybenzyl alcohol and acetic acid, but that these metabolites do not interfere with the FRET assay. (**f**_**1**_,**f**_**2**_) Unlike wild-type (WT) HEK293 cells (**f**_**1**_), the PDE inhibitor IBMX (50 μM) raised levels of cAMP in HEK293-sAC cells, an effect blocked by 50 μM of the sAC inhibitor LRE1 (**f**_**2**_). All panels indicate the final equilibrium concentrations of test agents, here and in all subsequent figures.

Forskolin induced a substantially larger ΔFRET in HEK293-H188-C24 cells as compared with clonal cell lines that instead expressed the earlier generation cAMP biosensors H74^37^, CEPAC^38^, or Epac1-camps^39^ (Fig. 2g–j). Notably, the ΔFRET measured for H188 in response to a saturating concentration of forskolin (6 μM) exceed that of the first generation cAMP biosensor Epac1-camps by *ca*. 5.5-fold (*c*.*f*., Fig. 2g,j). Since Epac1 responds to Epac-selective cAMP analogs but not PKA-selective analogs^40^, we sought to determine if the truncation and mutation of Epac1 within H188 might render it incapable of differentiating between these two classes of cyclic nucleotides. This was not the case since the selective Epac activator 8-pCPT-2′-*O*-Me-cAMP-AM was detected by H188 (Fig. 3a_1_), whereas the selective PKA activator 6-Bnz-cAMP-AM was not (Fig. 3a_2_). Importantly, the ΔFRET measured in response to 8-pCPT-2′-*O*-Me-cAMP-AM was reduced by CE3F4^41^, a tetrahydroquinoline that is a specific inhibitor of Epac1 activation (Fig. 3b_1_). In contrast, ESI-05, a trimethylphenylsulfone that is a specific inhibitor of Epac2 activation^42^, was without effect (Fig. 3b_2_). Neither CE3F4 nor ESI-05 alone exerted a direct effect on the baseline FRET ratio (Fig. 3c_1_,c_2_).

The cAMP antagonist prodrug Rp-8-Br-cAMPS-pAB inhibited the ΔFRET measured in response to forskolin (Fig. 3d_1_), while also exerting a weak partial agonist effect (Fig. 3d_2_)^36^. These actions of Rp-8-Br-cAMPS-pAB were not reproduced by the negative control 4-acetoxybenzy alcohol (4-Abn-OH) that lacks the cAMP moiety (Fig. 3e_1_,e_2_)^36^. Finally, we expressed H188 in HEK293 cells by viral transduction to demonstrate that soluble adenylyl cyclase (sAC) activity could be monitored in FRET assays that compared wild-type (WT) HEK293 cells or HEK293-sAC cells treated with IBMX in the presence or absence of the selective sAC inhibitor LRE1 (Fig. 3f_1_,f_2_)^43,44^. Collectively, these findings demonstrated that H188 expressed in HEK293 cells served as an accurate biosensor for detection of cAMP.

### Evaluation of GLP-1R agonist action in assays of FRET

**Figure 4:**
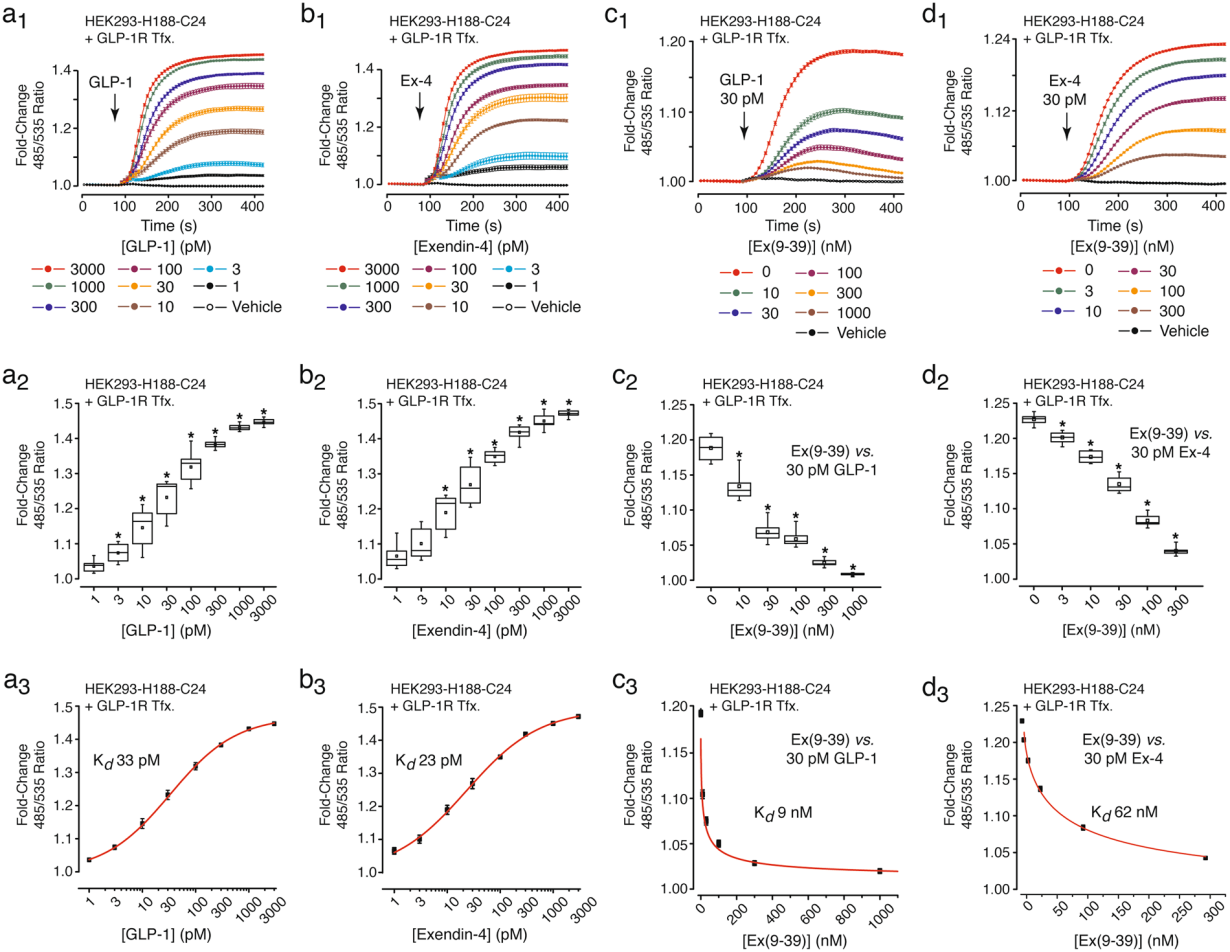
GLP-1R agonist action in HEK293-H188-C24 cells transfected with the GLP-1R. (**a**_**1**_,**b**_**1**_) Levels of cAMP increased in HEK293-H188-C24 cells transfected (Tfx.) with the human GLP-1R and treated with GLP-1 (**a**_**1**_) or exendin-4 (abbreviated as Ex-4) (**b**_**1**_). (**a**_**2**_,**b**_**2**_) Box-and-whisker plots summarizing the dose-dependent actions of GLP-1 (**a**_**2**_) or exendin-4 (**b**_**2**_). (**a**_**3**_,**b**_**3**_) Hill plots for GLP-1 (**a**_**3**_) and exendin-4 (**b**_**3**_). (**c**_**1**_,**d**_**1**_) Ex(9–39) blocked the actions of GLP-1 (**c**_**1**_) or exendin-4 (**c**_**2**_). (**c**_**2**_,**d**_**2**_) Box-and-whisker plots summarizing the dose-dependent ability of Ex(9–39) to antagonize the actions of GLP-1 (**c**_**2**_) or exendin-4 (d_2_) (**c**_**3**_,**d**_**3**_) Hill plot derivations of K_*d*_ values for Ex(9–39) in assays using GLP-1 (**c**_**3**_) or exendin-4 (**d**_**3**_). *Indicates a *P* value of <0.01, one-way ANOVA with post-hoc Tukey. Comparisons in **a**_**2**_ and **b**_**2**_ are between cells not treated (vehicle control) or treated with the indicated concentrations of GLP-1R agonists. Comparisons in **c**_**2**_ and **d**_**2**_ are between cells treated with GLP-1R agonists in the absence or the presence of the indicated concentrations of Ex(9–39).

Prior to screening for an effect of EP45 at the GLP-1R, we validated that this FRET assay was able to detect stimulatory effects of established GLP-1R agonists. To this end, HEK293-H188-C24 cells were transfected with the human GLP-1R^45^, after which GLP-1 or exendin-4 were administered to monolayers of cells grown in individual wells of a 96-well plate. H188 detected the dose-dependent cAMP-elevating actions of GLP-1 (Fig. 4a_1_–b_3_) and exendin-4 (Fig. 4b_1_–b_3_), whereas the GLP-1R antagonist exendin(9–39) blocked the agonist actions of GLP-1 (Fig. 4c_1_–c_3_) and exendin-4 (Fig. 4d_1_–d_3_). Apparent K_*d*_ values for GLP-1 and exendin-4 agonist action were 33 and 23 pM, respectively (Fig. 4a_3_,b_3_). K_*d*_ values for exendin(9–39) antagonist action versus GLP-1 and exendin-4 were 9 and 62 nM, respectively (Fig. 4c_3_,d_3_). GLP-1 and exendin-4 were without effect in cells transfected with the negative control empty vector (EV) (Suppl. Fig. 2a_1_,a_2_).

**Figure 5:**
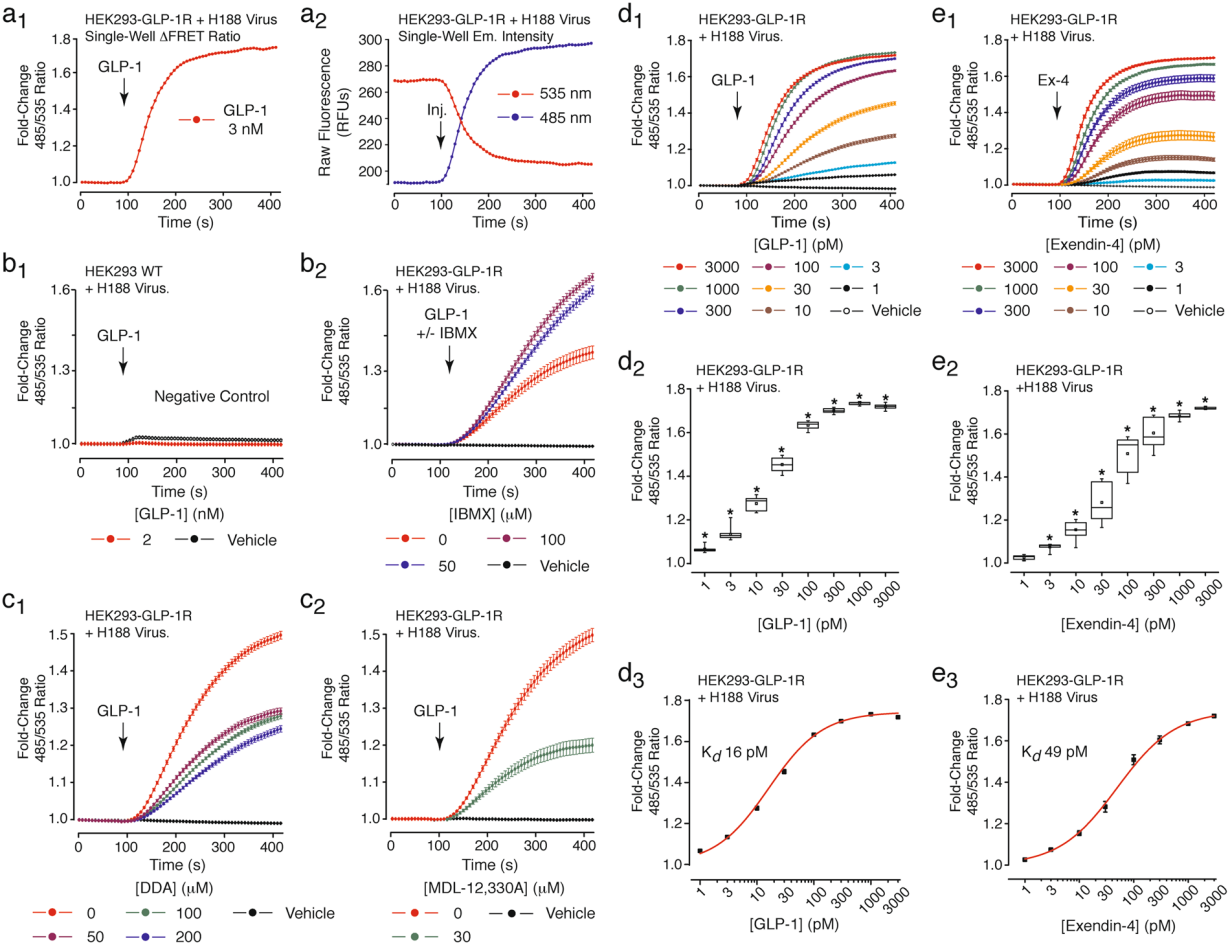
GLP-1R agonist action in HEK293-GLP-1R cells virally transduced with H188. (**a**_**1**_,**a**_**2**_) FRET-based single-well detection of GLP-1-stimulated cAMP production in HEK293-GLP-1R cells stably expressing the human GLP-1R and virally transduced with H188. (**b**_**1**_) Negative control illustrating no effect of GLP-1 in wild-type (WT) HEK293 cells transduced with H188 but lacking GLP-1 receptors. (**b**_**2**_) IBMX potentiated the action of GLP-1 in HEK293-GLP-1R cells transduced with H188. (**c**_**1**_,**c**_**2**_) DDA (**c**_**1**_) and MDL-12,330A (**c**_**2**_) suppressed the action of GLP-1 in HEK293-GLP-1R cells transduced with H188. (**d**_**1**_**–d**_**3**_) and (**e**_**1**_**–e**_**3**_) Dose-response, box-and-whisker, and Hill plots for GLP-1 (**d**_**1**_–**d**_**3**_) and exendin-4 (**e**_**1**_–**e**_**3**_) in HEK293-GLP-1R cells transduced with H188. *Indicates a *P* value of < 0.01, one-way ANOVA with post-hoc Tukey. Comparisons in **d**_**2**_ and **e**_**2**_ are between cells not treated (vehicle control) or treated with the indicated concentrations of GLP-1R agonists.

To simplify this assay so that transient transfection with the GLP-1R was not required, H188 was expressed by adenoviral transduction in HEK293-GLP-1R cells that stably expressed the human GLP-1R^46^. This approach allowed single well detection of the ΔFRET in response to GLP-1 (Fig. 5d_a_,d_2_), whereas no such effect of GLP-1 was measured when testing wild-type HEK293 cells transduced with the H188 virus (Fig. 5b_1_). As expected, the PDE inhibitor IBMX potentiated the action of GLP-1 (Fig. 5b_2_), whereas two selective inhibitors of transmembrane adenylyl cyclases (DDA, MDL-12,330 A) suppressed the action of GLP-1 (Fig. 5c_1_,c_2_). It was also possible to obtain accurate dose-response relationships describing the cAMP-elevating actions of GLP-1 (Fig. 5d_1_–d_3_ and exendin-4 (Fig. 5e_1_–e_3_) in HEK293-GLP-1R cells expressing H188. K_*d*_ values for GLP-1 and exendin-4 agonist action were 16 and 49 pM, respectively (Fig. 5d_3_,e_3_).

### EP45 is a potent and effective GLP-1R agonist

**Figure 6:**
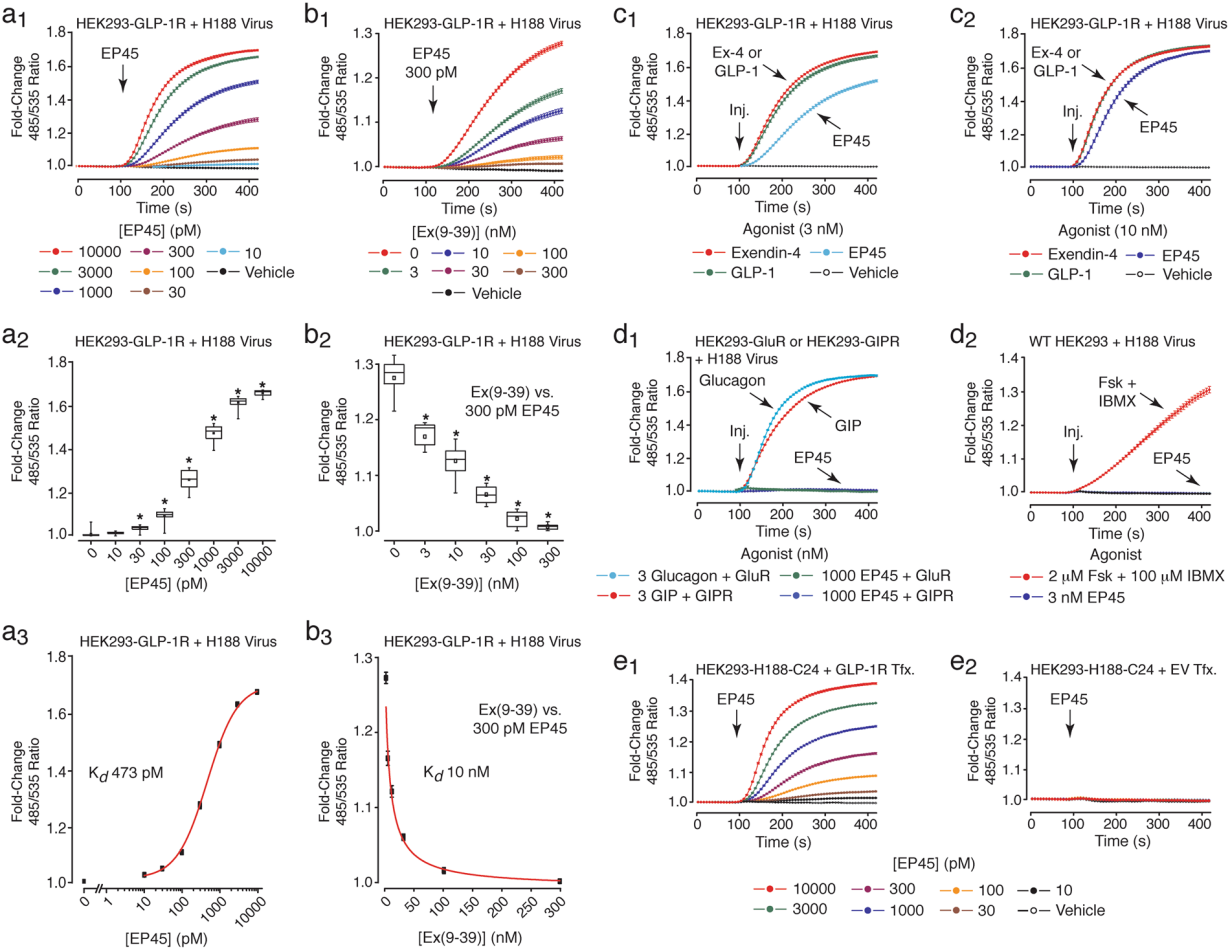
EP45 signals *via* the GLP-1R to stimulate cAMP production. (**a**_**1**_**–a**_**3**_) FRET data (**a**_**1**_), box-and-whisker plot (**a**_**2**_), and Hill plot (**a**_**3**_) summarizing findings in which EP45 (10–10,000 pM) exerted a dose-dependent action (K_*d*_ 473 pM) to increase levels of cAMP in HEK293-GLP-1R cells virally transduced with H188. (**b**_**1**_**–b**_**3**_) FRET data (**b**_**1**_), box-and-whisker plot (**b**_**2**_), and Hill plot (**b**_**3**_) summarizing findings in which the GLP-1R antagonist Ex(9–39) (3–300 nM) exerted a dose-dependent effect (K_*d*_ 10 nM) to block the action of EP45 (300 pM) in HEK293-GLP-1R cells transduced with H188. (**c**_**1**_,**c**_**2**_) HEK293-GLP-1R cells were transduced with H188 so that comparisons could be made concerning the potency and efficacy of GLP-1, exendin-4, and EP45 when each was tested at 3 nM (**c**_**1**_) or 10 nM (**c**_**2**_) concentrations. (**d**_**1**_) EP45 (1,000 nM) failed to raise levels of cAMP in HEK293 cells virally transduced with H188, while also stably transfected with glucagon or GIP receptors (GluR, GIPR). In contrast, Glucagon (3 nM) and GIP (3 nM) did raise levels of cAMP when each was tested in these same HEK293 cells expressing either glucagon or GIP receptors. (**d**_**2**_) Wild-type (WT) HEK293 cells virally transduced with H188 failed to respond to EP45, whereas they did respond to the cAMP-elevating agents forskolin and IBMX. (**e**_**1**_,**e**_**2**_) EP45 raised levels of cAMP in HEK293-H188-C24 cells transfected with the human GLP-1R (**e**_**1**_), but not in cells transfected with the negative control empty vector (EV) (**e**_**2**_). *Indicates a *P* value of < 0.01, one-way ANOVA with post-hoc Tukey. Comparisons in **a**_**2**_ are between cells not treated (vehicle control) or treated with the indicated concentrations of EP45. Comparisons in **b**_**2**_ are between cells treated with EP45 in the absence or the presence of the indicated concentrations of Ex(9–39).

There existed a dose-dependent action of EP45 (10–10,000 pM) to raise levels of cAMP in HEK293-GLP-1R cells virally transduced with H188 (Fig. 6a_1_–a_3_). This action of EP45 was blocked by the GLP-1R antagonist exendin(9–39) (Fig. 6b_1_–b_3_)^47^. K_*d*_ values for agonist and antagonist actions of EP45 and exendin(9–39) were 473 pM and 10 nM, respectively (Fig. 6a_3_,b_3_). When testing 3 nM EP45 the ΔFRET exhibited a slower time course and smaller end-point amplitude in comparison to GLP-1 and exendin-4 (Fig. 6c_1_). However, 10 nM EP45 more fully replicated the actions of GLP-1 and exendin-4 with respect to the time courses and amplitudes of the responses (Fig. 6c_2_). EP45 exhibited true GLP-1R specificity since it exerted no effect when tested at 1,000 nM in HEK293 cells that stably expressed rat glucagon or rat GIP receptors (Fig. 6d_1_). Furthermore, EP45 was without effect in wild-type HEK293 cells, although these cells responded to forskolin and IBMX (Fig. 6d_2_). Thus, our HEK293 cells did not express endogenous receptors for EP45. Additional control experiments demonstrated that EP45 raised levels of cAMP in HEK293-H188-C24 cells transfected with the human GLP-1R, but not in cells transfected with the negative control empty vector (Fig. 6e_1_,e_2_).

### EP45 is an effective NPY2R agonist

We also tested for the ability of EP45 to act as an NPY2R agonist and to alter levels of cAMP in FRET assays using HEK293-H188-C24 cells transfected with human NPY2R^48^. Our focus on cAMP signaling was prompted by the fact that EP45 had little ability to raise levels of Ca^2+^ in HEK293 cells transfected with NPY2R (Suppl. Fig. 3a–d). Since NPY2R signals through G_i_ proteins to inhibit adenylyl cyclase activity^48^, we predicted that an agonist action of EP45 at NPY2R to lower levels of cAMP would be measurable as a decrease of the baseline 485/535 nm FRET ratio. However, detection of such a ΔFRET was not feasible at resting levels of cAMP due to the fact that basal adenylyl cyclase activity in HEK293 cells was apparently low. In fact, IBMX (50 μM) failed to raise levels of cAMP in wild-type HEK293 cells (refer back to Fig. 3f_1_). Therefore, EP45 agonist action was instead monitored under conditions in which adenylyl cyclase activity was stimulated during treatment of HEK293 cells with forskolin. Our expectation was that this protocol would reveal the inhibitory effect of NPY2R agonists on cAMP production. To test this prediction, the FRET assay was optimized to detect the cAMP-lowering action of NPY2R agonist PYY(3–36), while also comparing its action to that of EP45.

**Figure 7:**
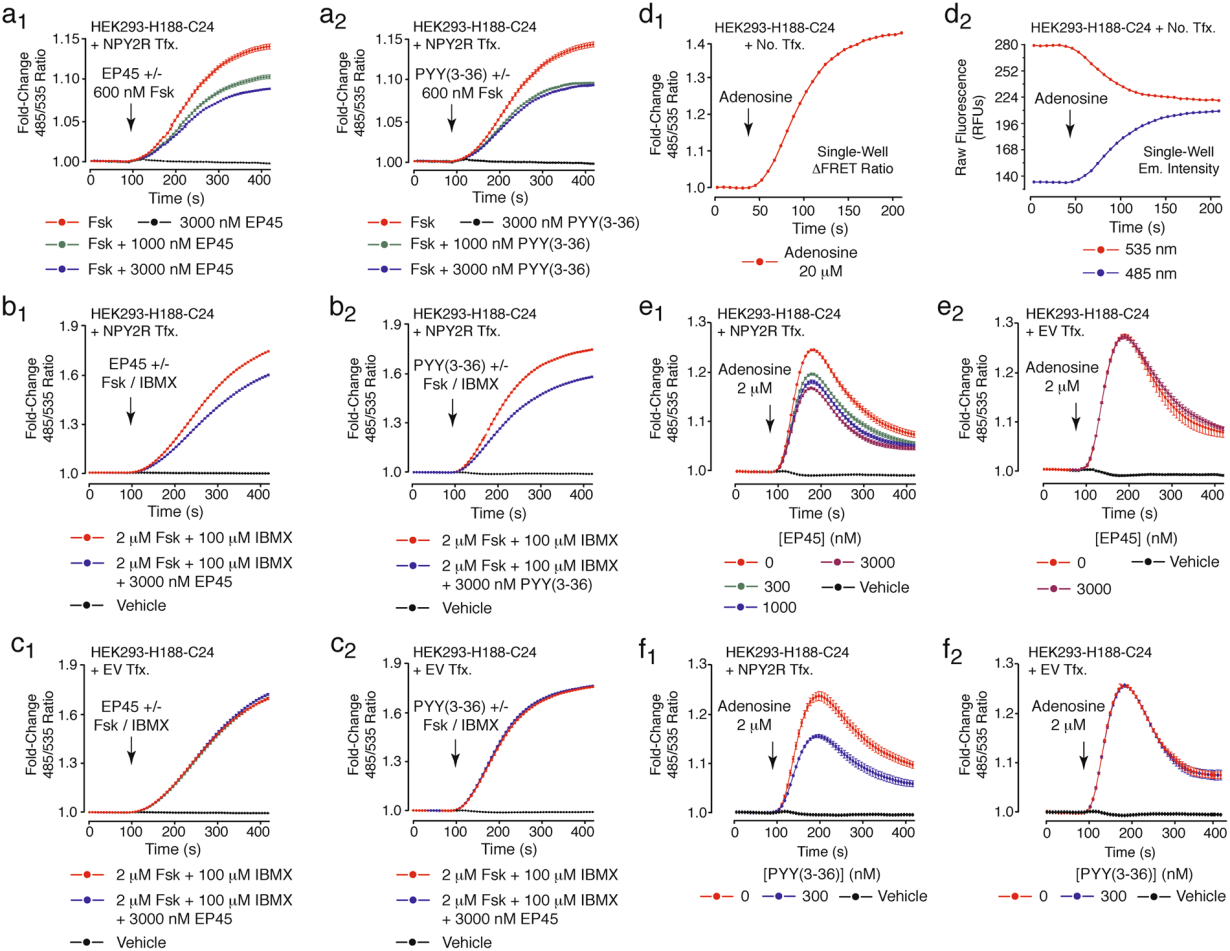
EP45 and PYY(3–36) signal *via* NPY2R to inhibit cAMP production. (**a**_**1**_,**a**_**2**_) Forskolin (Fsk, 600 nM) -stimulated cAMP production in HEK293-H188-C24 cells transfected with human NPY2R was reduced by co-administered EP45 (**a**_**1**_) or PYY(3–36) (**a**_**2**_). (**b**_**1**_,**b**_**2**_) Both EP45 (**b**_**1**_) and PYY(3–36) (**b**_**2**_) retained their abilities to inhibit forskolin (2 μM) -stimulated cAMP production in HEK293-H188-C24 cells transfected with NPY2R and treated with IBMX (100 μM). (**c**_**1**_,**c**_**2**_) Negative controls demonstrating that EP45 (**c**_**1**_) and PYY(3–36) (**c**_**2**_) failed to inhibit forskolin-stimulated cAMP production in HEK293-H188-C24 cells transfected with the empty vector (EV) and treated with IBMX. (**d**_**1**_,**d**_**2**_) FRET-based single-well detection of adenosine-stimulated cAMP production mediated by endogenous adenosine receptors in untransfected HEK293-H188-C24 cells. (**e**_**1**_,**e**_**2**_) EP45 inhibited adenosine-stimulated cAMP production in HEK293-H188-C24 cells transfected with NPY2R (**e**_**1**_) but not the negative control empty vector (**e**_**2**_). (**f**_**1**_,**f**_**2**_) PYY(3–36) also inhibited adenosine-stimulated cAMP production in HEK293-H188-C24 cells transfected with NPY2R (**f**_**1**_) but not the negative control empty vector (**f**_**2**_).

For HEK293-H188-C24 cells transfected with human NPY2R, there existed inhibitory actions of EP45 and PYY(3–36) to counteract the ability of forskolin (600 nM) to raise levels of cAMP (Fig. 7a_1_,a_2_). These actions of EP45 and PYY(3–36) resulted from functional antagonism of forskolin-stimulated cAMP production because the baseline FRET ratio was not altered when EP45 or PYY(3–36) were administered alone (Fig. 7a_1_,a_2_). Importantly, we considered the theoretical possibility that NPY2R agonists might lower levels of cAMP not simply by reducing adenylyl cyclase activity, but by instead increasing PDE activity. This alternative mechanism of action was unlikely since EP45 and PYY(3–36) retained their capacities to counteract stimulatory effects of forskolin (2 μM) when cells were treated with 100 μM of the PDE inhibitor IBMX (Fig. 7b_1_,b_2_). Under these conditions nearly identical findings were obtained when testing PYY(1–36), the extended form of PYY that acts as an agonist at both NPY2R and NPY1R (Suppl. Fig. 2b_1_)^49,50^. Importantly, no such inhibitory actions of EP45 and PYY(3–36) were measured when cells were transfected with the empty vector serving as a negative control (Fig. 7c_1_,c_2_).

### EP45 signals through NPY2R to suppress A2_B_ receptor-stimulated cAMP production

**Figure 8:**
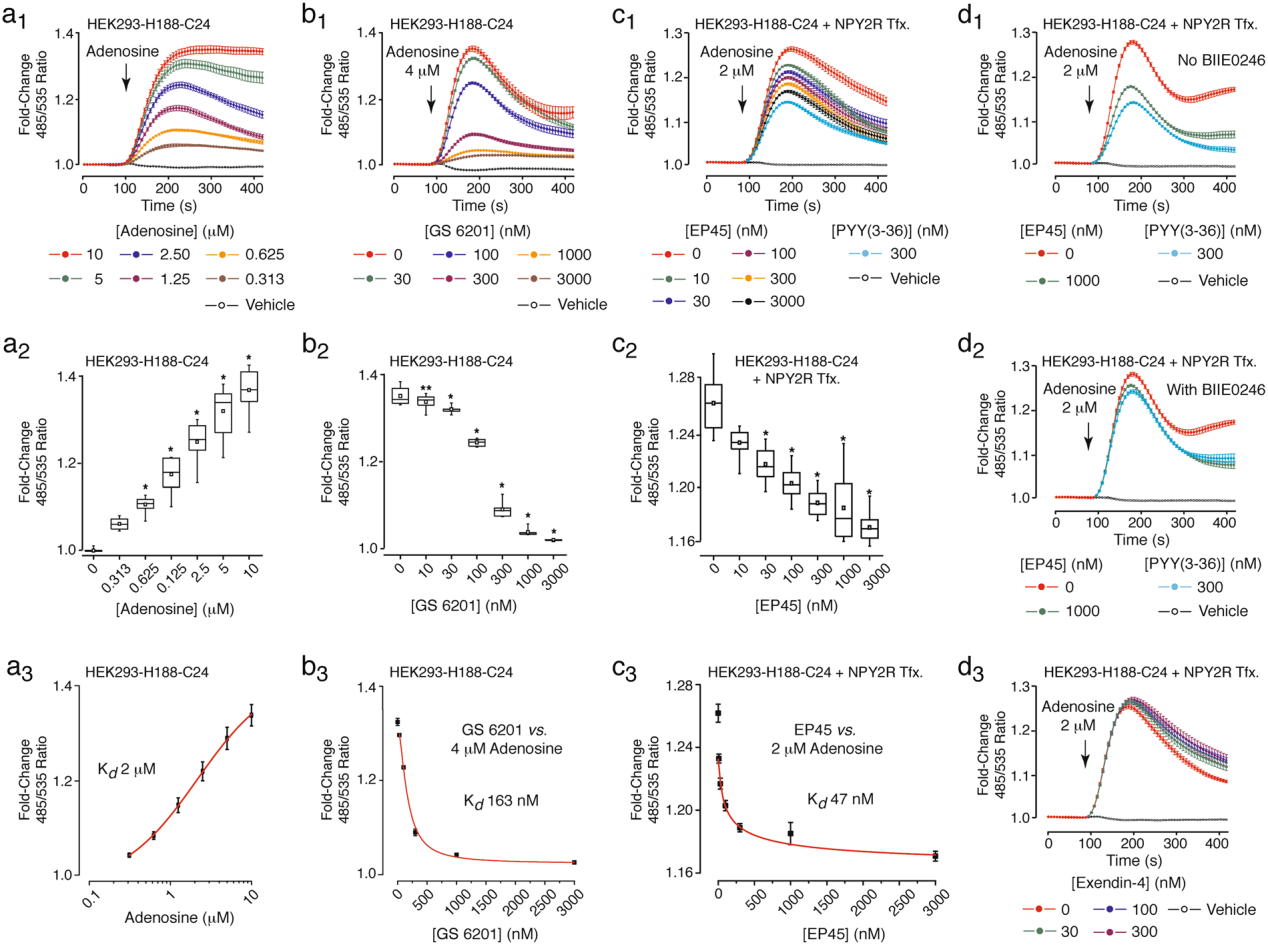
EP45 signals *via* NPY2R to inhibit A2_B_ receptor-stimulated cAMP production. (**a**_**1**_**–a**_**3**_) FRET data (**a**_**1**_), box-and-whisker plot (**a**_**2**_), and Hill plot (**a**_**3**_) summarizing findings in which adenosine (0.313–10 μM) exerted a dose-dependent action (K_*d*_ 2 μM) to increase levels of cAMP in HEK293-H188-C24 cells. (**b**_**1**_**–b**_**3**_) FRET data (**b**_**1**_), box-and-whisker plot (**b**_**2**_), and Hill plot (**b**_**3**_) summarizing findings in which the A2_B_ receptor antagonist GS 6201 exerted a dose-dependent effect (K_*d*_ 163 nM) to inhibit the cAMP-elevating action of adenosine (4 μM). (**c**_**1**_**–c**_**3**_) FRET data (**c**_**1**_), box-and-whisker plot (**c**_**2**_), and Hill plot (**c**_**3**_) summarizing findings from a single experiment in which EP45 exerted a dose-dependent effect (K_*d*_ 47 nM) to inhibit the cAMP-elevating action of adenosine (2 μM). (**d**_**1**_,**d**_**2**_) NPY2R antagonist BIIE0246 (300 nM) reduced the actions of EP45 and PYY(3–36) to suppress stimulatory effects of 2 μM adenosine. (**d**_**3**_) Exendin-4 (30–300 nM) failed to suppress the action of adenosine (2 μM) in HEK293-H188-C24 cells transfected with NPY2R. * or **Indicate *P* values of < 0.01 or < 0.05, respectively, one-way ANOVA with post-hoc Tukey. Comparisons in **a**_**2**_ are between cells not treated (vehicle control) or treated with the indicated concentrations of adenosine. Comparisons in **b**_**2**_ are between cells treated with adenosine in the absence or the presence of the indicated concentrations of GS6201. Comparisons in **c**_**2**_ are between cells treated with adenosine in the absence or the presence of the indicated concentrations of EP45.

We next tested to see if EP45 would signal through NPY2R to suppress stimulatory effects of a GPCR agonist on cAMP production. This prediction was tested using HEK293-H188-C24 cells transfected with human NPY2R and treated with the GPCR agonist adenosine that stimulates the endogenous adenosine A2_B_ receptors that signal through G_s_ proteins to stimulate adenylyl cyclase activity and to raise levels of cAMP in HEK293 cells^51^. Since NPY2R signals through G_i_ proteins to reduce adenylyl cyclase activity, it was our prediction that an NYP2R-mediated action of EP45 would be measurable as a suppression of adenosine-stimulated cAMP production. Consistent with this prediction, cAMP production was measured as an increase of the 485/535 nm H188 FRET ratio in HEK293-H188-C24 cells treated with adenosine (Fig. 7d_1_,d_2_). When HEK293-H188-C24 cells were transfected with human NPY2R, this action of adenosine (2 μM) was reduced in a dose-dependent manner by 300–3,000 nM EP45 (Fig. 7e_1_). However, no such action of EP45 was measured in cells transfected with the empty vector (Fig. 7e_2_). Similar findings were obtained when testing adenosine and PYY(3–36) using HEK293-H188-C24 cells transfected with NPY2R but not the empty vector (Fig. 7f_1_,f_2_). These observations prompted us to investigate the action of adenosine in greater detail so that its A2_B_ receptor-mediate action could be substantiated. Using HEK293-H188-C24 cells not transfected with NPY2R, the action of adenosine to raise levels of cAMP was first quantified by dose-response analysis (Fig. 8a_1_–a_3_), while also establishing that its cAMP-elevating action was blocked by the selective A2_B_ receptor antagonist GS6201 (Fig. 8b_1_–b_3_)^52^. K_*d*_ values for agonist and antagonist actions of adenosine and GS6201 were 2 μM and 163 nM, respectively (Fig. 8a_3_,b_3_).

Next, we quantified the NPY2R-mediated action of EP45 to inhibit adenosine-stimulated cAMP production. This analysis used a concentration of adenosine (2 μM) equivalent to its K_*d*_ value, as described above (Fig. 8a_3_). For HEK293-H188-C24 cells transfected with human NPY2R and treated with adenosine, dose-response analysis established that EP45 exerted a significant inhibitory effect within a concentration range of 10–3,000 nM (Fig. 8c_1_–c_3_). However, a comparison of the action of EP45 with that of PYY(3–36) revealed that the inhibitory effect of 300 nM PYY(3–36) exceed that of 3,000 nM EP45 (Fig. 8c_1_). Thus, the chimeric peptide EP45 has reduced agonist activity at NPY2R relative to that of native PYY(3–36). Still, the estimated K_*d*_ value derived for EP45 from this dose-response analysis was 47 nM (Fig. 8c_3_), whereas that calculated for PYY(3–36) was 18 nM (Suppl. Fig. 4a_1_–a_3_). It may be concluded that the K_*d*_ value calculated for EP45 is not incompatible with the potential use of EP45 as a prototype for future optimization of NPY2R agonist potency, efficacy, and selectivity. Importantly, BIIE0246 (300 nM), a selective NPY2R antagonist^53^, reduced the inhibitory actions of EP45 (1,000 nM) and PYY(3–36) (300 nM) when testing 2 μM adenosine in assays of HEK293-H188-C24 cells transfected with NPY2R (Fig. 8d_1_,d_2_). Finally, we took into account the theoretical possibility that the fragment of exendin-4 at amino acid residues 1–33 of EP45 might exert NPY2R agonist activity (Fig. 1). This possibility is unlikely since full-length exendin-4 (30–300 nM) failed to counteract adenosine-stimulated cAMP production in HEK293-H188-C24 cells transfected with human NPY2R (Fig. 8d_3_). Thus, the agonist action of EP45 at NPY2R was most likely conferred by the peptide’s C-terminal amino acid residues 34–45 that correspond to amino acid residues 25–36 located within the C-terminal of PYY(3–36) (Fig. 1).

## Discussion

Herein we demonstrate novel dual agonist properties of EP45, a rationally designed chimeric peptide that is predicted to stimulate the GLP-1R and NPY2R at multiple target organs. This discovery is validated using a new high-throughput FRET assay that itself provides a new platform for medicinal chemistry research. In these FRET assays, EP45 stimulates cAMP production via the GLP-1R, while it instead inhibits cAMP production via NPY2R. Such findings indicate that EP45 or similarly designed chimeric peptides might serve as novel dual agonists with potential blood glucose-lowering and/or appetite-suppressing properties that are of relevance to the treatment of T2DM and obesity.

Our approach to the design of EP45 took two routes, run in parallel. The first approach built from the core 34 residue PYY(3–36), attempting to incorporate key exendin-4 residues in a hybridization approach. All attempts in this direction failed to produce a dual agonist. The second approach built from the core 39 residue exendin-4, focusing on the C-terminal extended (relative to GLP-1) sequence of exendin-4 fused to varying lengths of the C-terminal region of PYY(3–36). This approach was taken given that PYY(3–36) is readily truncated or modified at the N-terminal region without loss of NPY2R agonism^54,55^, and that modification of the N-terminal region of exendin-4 produces antagonists of the GLP1-R^47^, yet the C-terminal hexapeptide is not critical for GLP1-R agonism^56^. The second approach ultimately produced EP45, a 45 residue monomeric peptide that is a true chimera of exendin-4 and PYY3–36. EP45 displays potent, concerted, independent agonism at both the GLP1-R and NPY2R. Although EP45 displays promiscuous agonism at the GLP1-R and NPY2R, it does not stimulate other structurally similar Class B GPCRs such as the GIPR or the glucagon receptor, and as such does not replicate the cAMP-elevating actions of GIP or glucagon.

The design of EP45 was inspired by prior research that led us to predict multiple benefits of a dual agonist peptide incorporating amino acid motifs found within exendin-4 and PYY(3–36). With respect to exendin-4, it is a GLP-1R agonist first isolated from the salivary glands of the lizard *Heloderma suspectum*^32^. It acts at the GLP-1R on beta cells of the endocrine pancreas to potentiate glucose-stimulated insulin secretion so that levels of blood glucose are lowered in patients with T2DM^28^. This antidiabetic action of exendin-4 is reinforced by its ability to upregulate insulin gene expression and biosynthesis, while also serving as a trophic factor so that beta cell growth, proliferation and survival are enhanced under conditions of T2DM^6^. Importantly, exendin-4 also acts at neuronal GLP-1 receptors^6^. Exendin-4 crosses the blood brain barrier to stimulate GLP-1 receptors located within the hypothalamus, and it also stimulates GLP-1 receptors located on vagal sensory neurons innervating the gut^6^. Such actions at neuronal GLP-1 receptors most likely explain the weight loss that is observed in some T2DM patients administered synthetic exendin-4 (exenatide)^6^.

PYY(3–36) is co-secreted with GLP-1 from enteroendocrine L-cells of the intestine^57^, and it too can cross the blood-brain barrier^10^. However, L-cells are not the exclusive sources of PYY(3–36) and GLP-1 since both peptides are synthesized by neurons located within key metabolic control centers of the central nervous system^6,10^. Appetite suppression that results from PYY(3–36) acting at hypothalamic NPY2R is abolished in NPY2R knockout mice^24^, or in wild-type mice in which PYY(3–36) is co-administered with an NPY2R antagonist^58^. Similarly, the anorectic action of GLP-1 is blocked by the GLP-1R antagonist exendin(9–39) when both agents are administered by intracerebroventricular injection^59^. Such prior findings suggest that it might be possible to adopt a dual agonist strategy to target both the GLP-1R and NPY2R simultaneously so that enhanced weight loss is achieved.

Intriguing are reports that PYY(3–36) stimulates GLP-1 release from L-cells^60^, whereas levels of circulating PYY(3–36) are reduced in obese individuals, but raised after exercise^61,62^. These findings have prompted interest in a potential usefulness of NPY2R agonists as a hormone replacement therapy for the treatment of obesity. However, the variability or weak efficacy of NPY2R agonists to suppress appetite remains a significant roadblock to their development as anti-obesity agents^10^. This roadblock might be explained by a physiological requirement for co-agonist activity of PYY(3–36) with hormones or neurotransmitters such as GLP-1 that participate in the control of appetite. In fact, exendin-4 and PYY(3–36) synergistically inhibit appetite in humans^63^, whereas combined infusion of exendin-4 and PYY(3–36) to mice results in a strong synergistic reduction of *ad libitum* calorie intake in comparison to monoinfusion of either peptide^64^. A synergistic reduction of food intake also occurs when rats with RYGB gastric bypass surgery are administered exendin-4 and PYY(3–36)^65^. Such findings have inspired a new field of combination drug therapy in which GLP-1R and NPY2R agonists are under investigation for use in the treatment of obesity^10^.

Dual GLP-1R/NPY2R agonists might be expected to satisfy the co-agonist dependence of appetite suppression whereby GLP-1 and PYY(3–36) synergistically regulate brainstem appetite control centers. If so, dual GLP-1R/NPY2R agonists might exert a less variable and more efficacious anorectic effect in comparison to GLP-1 or PYY(3–36) administered alone. Superimposed on this anorectic effect would be the action of dual GLP-1R/NPY2R agonists to lower levels of blood glucose. Thus, such dual agonists might provide a monotherapy with a constellation of highly desired metabolic actions relevant to the treatment of T2DM and obesity. For these reasons, there exists a strong rationale to evaluate potential synergistic outcomes of dual GLP-1R/NPY2R agonists in assays that monitor food intake, body weight, energy expenditure, and glycemia.

It should be noted that the FRET assay reported here represents a significant technical advance. We have established a high-throughput means with which to assess GPCR agonist or antagonist action using a clonal HEK293-H188-C24 cell line, or adenovirus that allow viral transduction of HEK293 cells with H188. These two approaches take advantage of the expanded dynamic range (2-fold ΔFRET) that H188 exhibits in comparison to earlier FRET reporters that were used in microplate reader assays as readouts for cAMP signaling^31,34,66^. For purposes of GPCR agonist screening, the accuracy and ease of FRET data acquisition in the high-throughput mode is preferable to live-cell imaging techniques using H188^30,67^, since the plate reader assay allows for a less variable population response in monolayers of cells. In fact, we find that it is possible to perform dose-response analyses so that apparent K_*d*_ values are obtained quickly when testing peptides such as GLP-1, exendin-4, and EP45.

It is also important to note that we find this high-throughput FRET assay to be applicable to the characterization of pharmacological agents that modify the catalytic activities of soluble adenylyl cyclase, transmembrane adenylyl cyclases, and cyclic nucleotide phosphodiesterases. Furthermore, we find that it is possible to monitor how synthetic cyclic nucleotide analogs act as agonists (8-pCPT-2′-*O*-Me-cAMP-AM) or antagonists (Rp-8-Br-cAMPS-pAB) when they interact with the cyclic nucleotide-binding (CNBD) domain of Epac1 contained within H188. Finally, we have established that this assay enables pharmacological characterization of small molecules such as CE3F4 and ESI-05 that act as specific inhibitors of Epac1 or Epac2, respectively. Thus, this microplate reader high-throughput FRET assay enables GPCR agonist/antagonist drug discovery, while also providing a new platform with which to advance cyclic nucleotide research.

## Methods

### Cell culture

The parental HEK293 cell line was obtained from the American Type Culture Collection (ATCC, Manassas, VA). HEK293 cells stably expressing the human GLP-1R were obtained from Novo Nordisk A/S (Bagsvaerd, Denmark)^46^. HEK293 cells stably expressing the GlucR or GIPR were obtained from C.G. Unson (Rockefeller University) or T.J. Kieffer (University of British Columbia), respectively^68^. HEK293 cells stably expressing H188, H74, CEPAC, or Epac1-camps were generated by O.G. Chepurny in the Holz laboratory. All cell cultures were maintained in Dulbecco’s Modified Eagles Medium (DMEM) containing 25 mM glucose and supplemented with 10% fetal bovine serum (FBS) and 1% penicillin-streptomycin. These cell cultures were equilibrated at 37 °C in a humidified incubator that was gassed with 5% CO_2_, and were passaged once a week. Culture media and additives were obtained from Thermo Fisher Sci. (Waltham, MA).

### Cell transfection

Transient transfections were performed using Lipofectamine and Plus Reagent (Thermo Fisher) using methodology described previously by our laboratory for HEK293 cells^68^. Plasmid DNA encoding the human GLP-1 receptor in pcDNA1 was obtained from M. Beinborn (Tufts Medical Center, Boston, MA)^45^. Plasmid encoding human NPY2R (I.D. NPYR20TN00) in pcDNA3.1 was from the University of Missouri-Rolla cDNA Resource Center (Rolla, MO). Individual clones of HEK293 cells stably expressing select FRET reporters were obtained by G418 antibiotic resistance selection, as described previously^34^. Adenovirus for transduction of HEK293 cells with H188 was generated by a commercial vendor (ViraQuest, North Liberty, IA) using the shuttle vector pVQAd CMV K-NpA and the H188 plasmid originally created by Klarenbeek and co-workers^30^.

### Assessment of H188 subcellular localization in HEK293-H188-C24 cells

Live-cell imaging to detect H188 subcellular localization was performed at 23 °C using a Zeiss Axio Observer.Z1 microscope equipped with 100x or 63x Plan-Apochromat Oil DIC objectives (Carl Zeiss, Inc., Thornwood, NY) and interfaced with a Hamamatsu Orca-Flash4.0 LT camera (Hamamatsu Photonics, Bridgewater, NJ), a PhotoFluor LM-75 light source (89 NORTH, Burlington, VT), and an FITC filter set (Chroma Technology Corp.). For co-detection of H188, DNA and F-actin in fixed and permeabilized HEK293-H188-C24 cells, the FITC filter set for detection of H188 was combined with a DAPI filter set for detection of DNA, and a TRITC filter set for detection of rhodamine phalloidin (Sigma-Aldrich, St. Louis, MO) that binds F-actin. MetaMorph imaging software (Molecular Devices) was used to control illumination shutters, camera exposure, and image acquisition. Image stacks were recorded with a Z-distance of 0.1 μm and subjected to deconvolution for construction of major projection overlays.

### Microscopy-based FRET reporter assay

HEK293-H188-C24 cells were plated onto glass coverslips mounted in a PDMI-2 micro-incubator (Warner Inst. Hamden, CT) for perifusion with buffer containing the test solutions. Live-cell imaging was performed using a Nikon Eclipse Ti-E inverted microscope equipped with a TIRF 60x objective (Micro Video Inst., Avon, MA), a Cascade 512b EMCCD camera (Photometrics, Tucson, AZ), and a chameleon-2 filter set (Chroma Technology Corp., Bellows Falls, VT) comprised of a D440/20 excitation filter, a 455DCLP dichroic, and D485/40 (CFP) or D535/30 (YFP) emission filters^34,35^. The excitation light source was a DeltaRam X monochromator (Photon Technology Int., Birmingham, NJ). Ratiometric analysis of the emitted light corresponding to fluorescence originating within a defined region of the cytoplasm was performed using Metafluor v.7.5 software (Molecular Devices).

### FRET reporter assay in a 96-well format

HEK293-H188-C24 cells stably expressing H188 were plated at 80% confluence on 96-well clear-bottom assay plates (Costar 3904, Corning, NY) coated with rat tail collagen (Collaborative Biomedical Products, Bedford, MA). HEK293 cells stably expressing select GPCRs were transduced for 16 hours with H188 virus at a density of *ca*. 60,000 cells/well under conditions in which the multiplicity of infection (m.o.i.) was equivalent to 25 viral particles per cell. For all assays, the culture media was then removed and replaced by 200 μl/well of a standard extracellular saline (SES) solution supplemented with 11 mM glucose and 0.1% BSA^31,34–36^. The composition of the SES was (in mM): 138 NaCl, 5.6 KCl, 2.6 CaCl_2_, 1.2 MgCl_2_, 11.1 glucose, and 10 Hepes (295 mOsmol, pH 7.4). Real-time kinetic assays of FRET were performed using a FlexStation 3 microplate reader equipped with monochromators (Molecular Devices, Sunnyvale, CA). Excitation light was delivered at 435/9 nm (455 nm cut-off), and emitted light was detected at 485/15 nm (CFP) or 535/15 nm (YFP)^31,34–36^. The emission intensities were the averages of 12 excitation flashes for each time point per well. Test solutions dissolved in SES were placed in V-bottom 96-well plates (Greiner Bio-One, Monroe, NC), and an automated pipetting procedure was used to transfer 50 μl of each test solution to each well of the assay plate containing monolayers of these cells. The 485/535 emission ratio was calculated for each well and the mean +/− *s*.*d*. values for 12 wells were averaged. To facilitate comparisons amongst different experiments, these FRET ratio values were normalized using baseline subtraction so that a y-axis value of 1 corresponds to the initial baseline FRET ratio, whereas a value of 2 corresponds to a doubling of the FRET ratio. The time course of the ΔFRET ratio was plotted after exporting data to Origin 8.0 (OriginLab, Northampton, MA). Origin 8.0 was also used for generation of Hill plots describing dose-response relationships.

### Fura-2 assay for detection of cytosolic [Ca^2+^]

HEK293 cell monolayers transfected with either NPY2R or the EV were loaded with the fluorescent Ca^2+^ indicator Fura-2-AM so that real-time kinetic assays of cytosolic [Ca^2+^] could be performed in a 96-well format using a Flexstation 3 microplate reader, as described previously^28^.

### Statistical analyses

The repeatability of findings was confirmed by performing each experiment a minimum of three times. FRET ratio data were evaluated for statistical significance by a one-way ANOVA test with a post-hoc Tukey HSD test. Comparisons of individual data sets are defined in the accompanying figure legends. A *P* value of < 0.05 was considered to be statistically significant.

### Sources of reagents

EP45 was produced by Genscript (NJ, USA) at ≥ 97% purity and with C-terminal amidation. Purity and identity were confirmed in-house by reversed-phase high-performance liquid chromatography (RP-HPLC) and electron spray mass spectroscopy (ESMS), respectively. All cAMP analogs were from the BIOLOG Life Science Institute (Bremen, Germany). GLP-1, glucagon, exendin-4, exendin(9–39), GIP, PYY(1–36), PYY(3–36), forskolin, IBMX, adenosine, DDA, MDL-12,330 A, carbachol, ATP, 4-acetoxybenzyl alcohol (4-Abn-OH), and ESI-05 were from Sigma-Aldrich (St. Louis, MO). CE3F4, GS6201, and BIIE0246 were from Tocris Biosciences (Minneapolis, MN). LRE1 and HEK293 cells stably expressing recombinant sAC were from Profs. L. R. Levin and J. Buck (Weill Cornell Medical College, New York, NY).

### Data availability

The data that support the findings of this study are available from the corresponding authors upon reasonable request.

## Electronic supplementary material

Supplementary Material
