# Publisher Correction: Chimeric peptide EP45 as a dual agonist at GLP-1 and NPY2R receptors

**DOI:** 10.1038/s41598-018-24359-2

**Published:** 2018-04-13

**Authors:** Oleg G. Chepurny, Ron L. Bonaccorso, Colin A. Leech, Torsten Wöllert, George M. Langford, Frank Schwede, Christian L. Roth, Robert P. Doyle, George G. Holz

**Affiliations:** 1 Department of Medicine, State University of New York (SUNY) Upstate Medical University, 505 Irving Avenue, Syracuse, NY 13210 USA; 2 Department of Chemistry, Syracuse University, 111 College Place, Syracuse, NY 13244 USA; 3 Department of Biology, Syracuse University, Syracuse, NY 13244 USA; 4 BIOLOG Life Science Institute, 28199 Bremen, Germany; 5 Center for Integrative Brain Research, Seattle Children’s Research Institute, Washington, 98105 USA; 6 Department of Pediatrics, University of Washington, Seattle, Washington, 98105 USA; 7 Department of Pharmacology, State University of New York (SUNY) Upstate Medical University, 505 Irving Avenue, Syracuse, NY 13210 USA

Correction to: *Scientific Reports* 10.1038/s41598-018-22106-1, published online 28 February 2018

In the original HTML version of this Article, an XML error led to incomplete Figure citations. This error has now been corrected in the HTML version; the PDF version was correct at the time of publication.

