# Supplementary material for: Chimeric peptide EP45 as a dual agonist at GLP-1 and NPY2R receptors
Source: Sci Rep. 2018 Feb 28;8:3749. doi: 10.1038/s41598-018-22106-1 (PMC5830615; doi:10.1038/s41598-018-22106-1)
Supplement: Supplementary file 1 — Supplementary Material [file 41598_2018_22106_MOESM1_ESM.pdf]

Supplementary Information

Chimeric peptide EP45 as a dual agonist at GLP-1 and NPY2R receptors

Oleg G. Chepurny†[1], Ron L. Bonaccorso†[2], Colin A. Leech[1], Torsten Wöllert[3], George M. Langford[3], Frank Schwede[4], Christian L. Roth[5,6], Robert P. Doyle[*1,2] & George G. Holz[*1,8]

Departments of Medicine[1] and Pharmacology[8]
State University of New York (SUNY) Upstate Medical University
505 Irving Avenue, Syracuse, NY 13210 (USA)

Department of Chemistry[2]
111 College Place
Syracuse University, Syracuse, NY 13244 (USA)

Department of Biology[3]
Syracuse University, Syracuse, NY 13244 (USA)

BIOLOG Life Science Institute[4]
28199 Bremen Germany

Center for Integrative Brain Research, Seattle Children's Research Institute[5]
Department of Pediatrics[6]
University of Washington, Seattle, Washington 98105 (USA)

Corresponding Authors[*]
George G. Holz (e-mail: holzg@upstate.edu)
Robert P. Doyle (e-mail: rpdoyle@syr.edu)

† Contributed equally to this work.

Running Title: *Dual agonist properties of EP45*

Supplementary Figure Legends

Figure S1. *In vitro* calibration of H188 FRET reporter cAMP sensitivity.

HEK293 cells grown on glass coverslips for live-cell fluorescence microscopy were transduced with H188 adenovirus on day-one of the assay, after which they were permeabilized with digitonin (10 μM) on day-two to allow access of extracellular cAMP to the cytosol. Using a live-cell imaging chamber and bath superfusion, it was possible to expose cells to known concentrations of cAMP so that the responsiveness of H188 could be calibrated using ratiometric imaging, as described in the main Methods section. Note that an *ca*. 2-fold ΔFRET was monitored over a cAMP concentration range that spanned 3 - 1,000 μM. Results depict the average time course of the ΔFRET for $n = 9$ cells. Horizontal bars indicate the time periods during which the bath superfusate contained the indicated concentrations of cAMP.

Figure S2. Transfection experiments using empty vector, NPY2R, and NPY1R.

(a_1, a_2) HEK293-H188-C24 cells transfected (Tfx.) with the negative control empty vector (EV) failed to respond to 10 nM GLP-1 (a_1) or 10 nM exendin-4 (a_2) in plate reader assays of FRET. **(b_1)** HEK293-H188-C24 cells transfected with human NPY2R were responsive to PYY(1-36) in the FRET assay, as determined by the ability of PYY(1-36) (3,000 nM) to counteract stimulatory effects of forskolin (2 μM) and IBMX (100 μM) on cAMP production.

Continued on the following page -

Figure S3. EP45 has little capacity to raise the cytosolic [Ca^{2+}].

(a) The viability of HEK293 cells transfected with human NPY2R was established in a fura-2 based Ca^{2+} assay in which endogenous muscarinic cholinergic and purinergic receptors were stimulated with the Ca^{2+} mobilizing agents carbachol or ATP, respectively. (**b**) Administration of EP45, PYY(1-36), or PYY(3-36) revealed that in comparison to carbachol and ATP, these agents do not effectively increase cytosolic [Ca^{2+}] in HEK239 cells transfected with NPY2R. (**c**) Expansion of the *y-axis* scaling confirmed the especially weak abilities of EP45, PYY(1-36), and PYY(3-36) to stimulate an increase of [Ca^{2+}] in HEK293 cells transfected with NPY2R. (**d**) None of these tested peptides altered the cytosolic [Ca^{2+}] in HEK239 cells transfected with the negative control empty vector. For each panel, the *y-axis* units indicate baseline-subtracted values of the absolute change of fura-2 ratio. Inj., injection of test agent.

Figure S4. Dose-response analysis for PYY(3-36) inhibitory action at NPY2R.

(a_1-a_3) Dose-response data (a_1), box-and-whisker plot (b_2), and Hill plot (b_3) summarizing findings from a single experiment in which PYY(3-36) exerted a dose-dependent effect (K_d 18 nM) to inhibit the cAMP-elevating action of adenosine (2 μM) in HEK293-H188-C24 cells transfected with NPY2R.

Figure S1

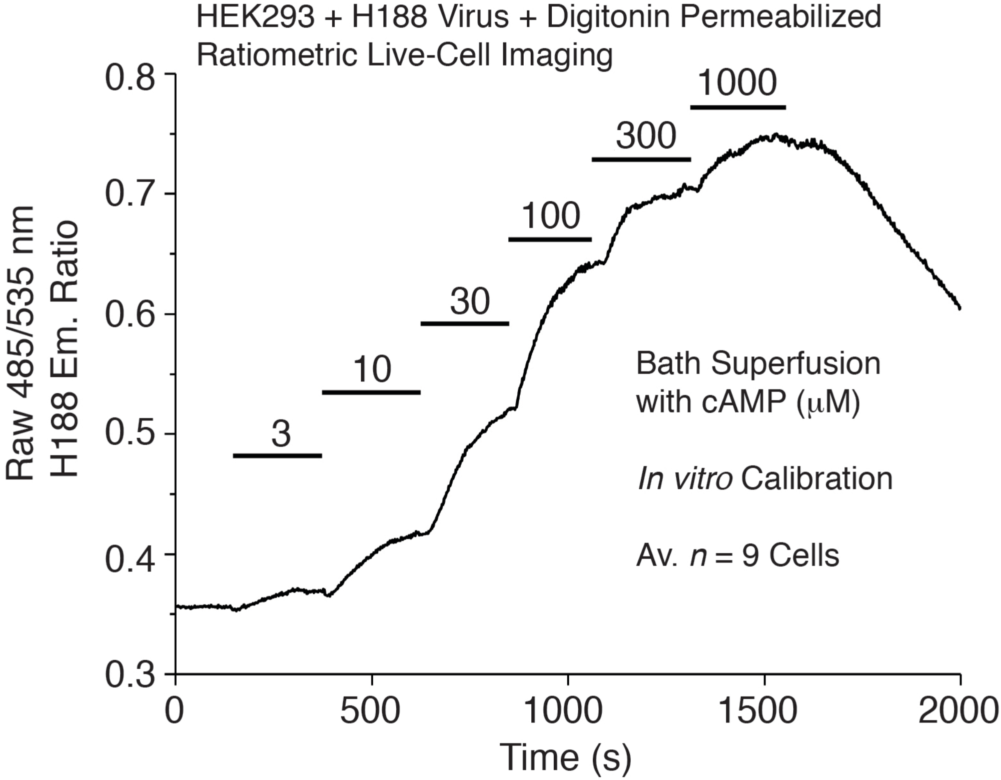

HEK293 + H188 Virus + Digitonin Permeabilized
Ratiometric Live-Cell Imaging
0.8
0.7
0.6
0.5
0.4
0.3
Raw 485/535 nm
H188 Em. Ratio
3
10
30
100
300
1000
Bath Superfusion
with cAMP (μM)
In vitro Calibration
Av. n = 9 Cells
0
500
1000
1500
2000
Time (s)

Figure S2

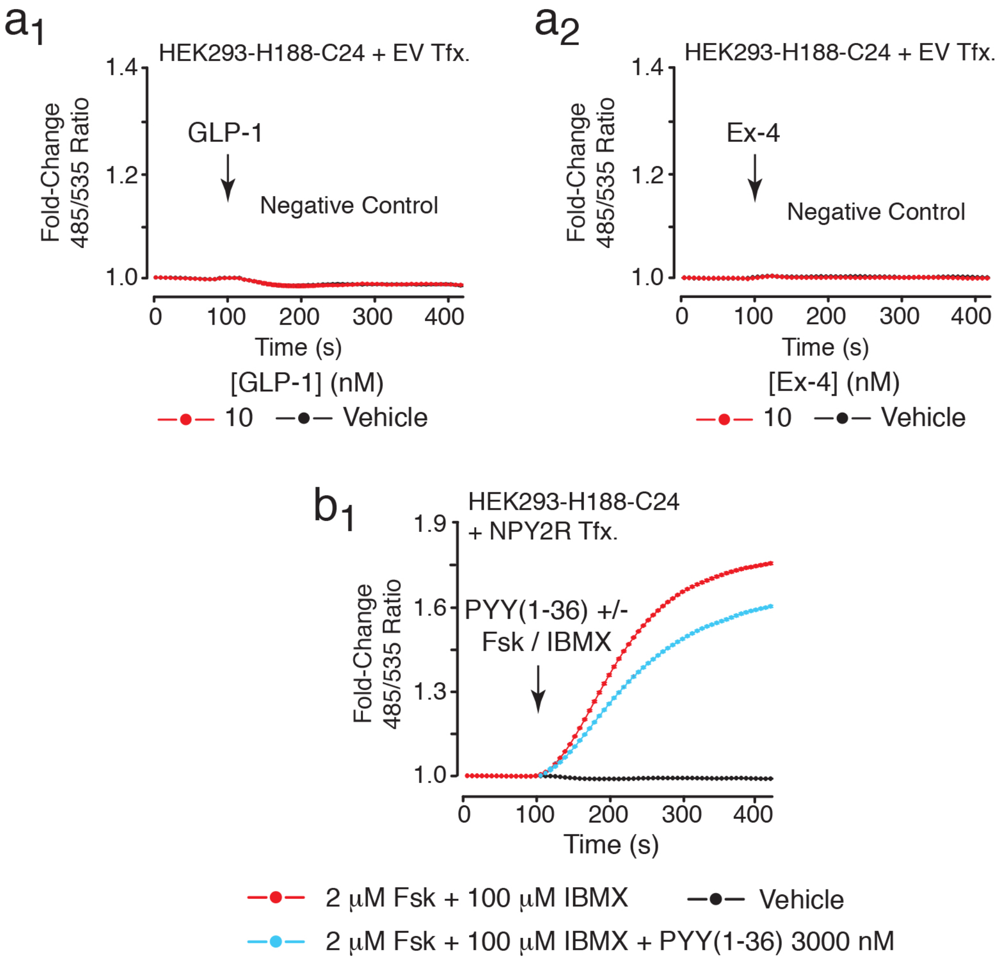
a1
HEK293-H188-C24 + EV Tfx.
Fold-Change 485/535 Ratio
1.4
1.2
1.0
GLP-1
Negative Control
0
100
200
300
400
Time (s)
[GLP-1] (nM)
10
Vehicle
a2
HEK293-H188-C24 + EV Tfx.
Fold-Change 485/535 Ratio
1.4
1.2
1.0
Ex-4
Negative Control
0
100
200
300
400
Time (s)
[Ex-4] (nM)
10
Vehicle
b1
HEK293-H188-C24 + NPY2R Tfx.
Fold-Change 485/535 Ratio
1.9
1.6
1.3
1.0
PYY(1-36) +/- Fsk / IBMX
0
100
200
300
400
Time (s)
2 μM Fsk + 100 μM IBMX
Vehicle
2 μM Fsk + 100 μM IBMX + PYY(1-36) 3000 nM

Figure S3

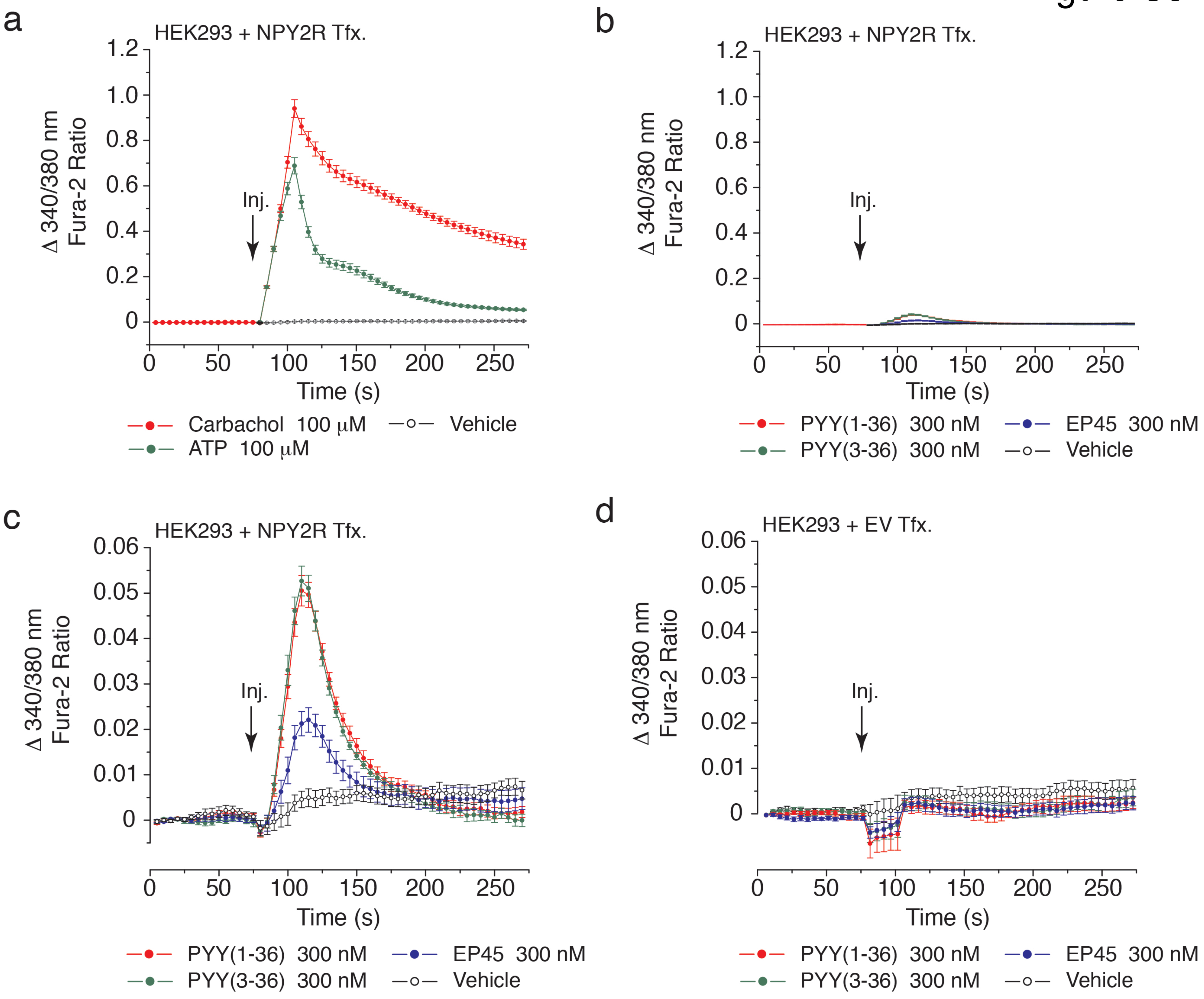
a
HEK293 + NPY2R Tfx.
Δ 340/380 nm Fura-2 Ratio
Inj.
Time (s)
Carbachol 100 μM
ATP 100 μM
Vehicle
b
HEK293 + NPY2R Tfx.
Δ 340/380 nm Fura-2 Ratio
Inj.
Time (s)
PYY(1-36) 300 nM
PYY(3-36) 300 nM
EP45 300 nM
Vehicle
c
HEK293 + NPY2R Tfx.
Δ 340/380 nm Fura-2 Ratio
Inj.
Time (s)
PYY(1-36) 300 nM
PYY(3-36) 300 nM
EP45 300 nM
Vehicle
d
HEK293 + EV Tfx.
Δ 340/380 nm Fura-2 Ratio
Inj.
Time (s)
PYY(1-36) 300 nM
PYY(3-36) 300 nM
EP45 300 nM
Vehicle

Figure S4

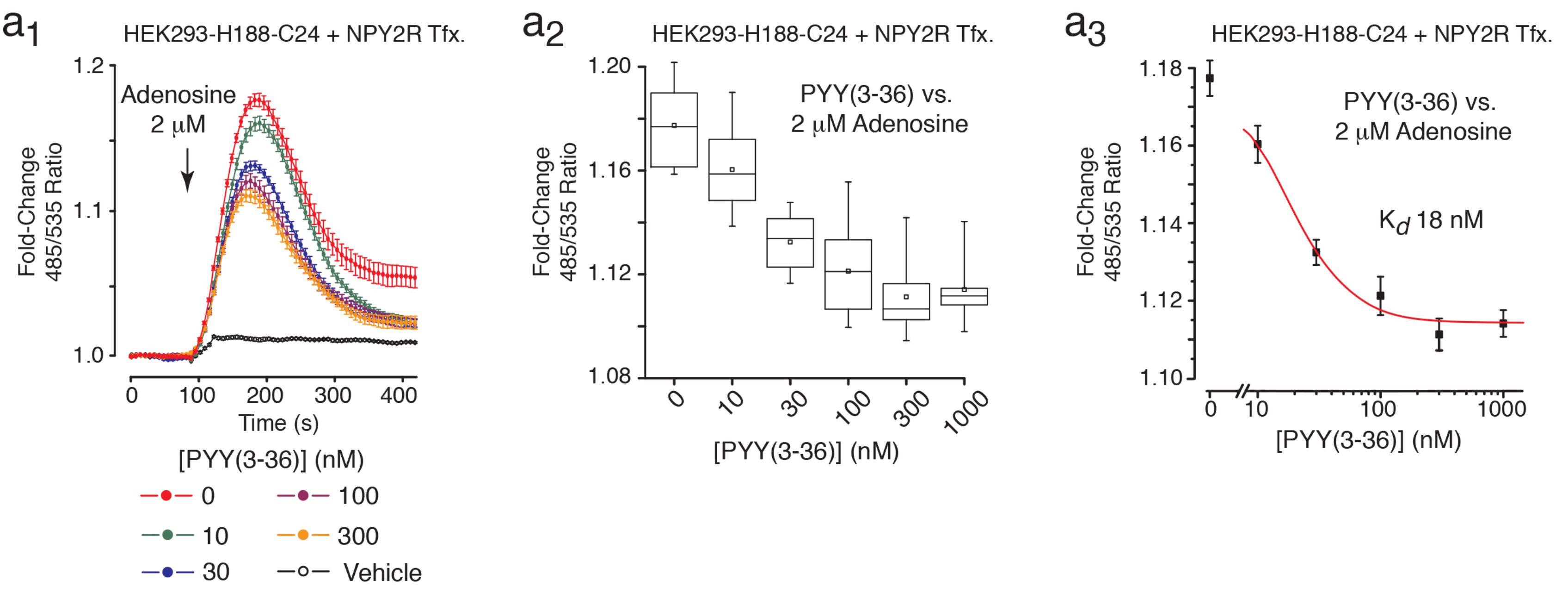
a1
HEK293-H188-C24 + NPY2R Tfx.
Adenosine
2 μM
Fold-Change
485/535 Ratio
1.2
1.1
1.0
0
100
200
300
400
Time (s)
[PYY(3-36)] (nM)
0
10
30
100
300
Vehicle
a2
HEK293-H188-C24 + NPY2R Tfx.
PYY(3-36) vs.
2 μM Adenosine
1.20
1.16
1.12
1.08
0
10
30
100
300
1000
[PYY(3-36)] (nM)
a3
HEK293-H188-C24 + NPY2R Tfx.
PYY(3-36) vs.
2 μM Adenosine
Kd 18 nM
1.18
1.16
1.14
1.12
1.10
0
10
100
1000
[PYY(3-36)] (nM)
